# Remote ischemic preconditioning of cardiomyocytes inhibits the mitochondrial permeability transition pore independently of reduced calcium‐loading or *sarc*K_ATP_ channel activation

**DOI:** 10.14814/phy2.12231

**Published:** 2014-11-26

**Authors:** Helen E. Turrell, Chokanan Thaitirarot, Hayley Crumbie, Glenn Rodrigo

**Affiliations:** 1 Department of Cardiovascular Sciences, University of Leicester, Glenfield General Hospital, Leicester, UK

**Keywords:** Ca^2+^‐Loading, ischemic preconditioning, MPT pore, remote ischemic preconditioning, sodium/hydrogen exchanger

## Abstract

Ischemic preconditioning (IPC) inhibits Ca^2+^‐loading during ischemia which contributes to cardioprotection by inhibiting mechanical injury due to hypercontracture and biochemical injury through mitochondrial permeability transition (MPT) pores during reperfusion. However, whether remote‐IPC reduced Ca^2+^‐loading during ischemia and its subsequent involvement in inhibiting MPT pore formation during reperfusion has not been directly shown. We have developed a cellular model of remote IPC to look at the impact of remote conditioning on Ca^2+^‐regulation and MPT pore opening during simulated ischemia and reperfusion, using fluorescence microscopy. Ventricular cardiomyocytes were isolated from control rat hearts, hearts preconditioned with three cycles of ischemia/reperfusion or naïve myocytes remotely conditioned with effluent collected from preconditioned hearts. Both conventional‐IPC and remote‐IPC reduced the loss of Ca^2+^‐homeostasis and contractile function following reenergization of metabolically inhibited cells and protected myocytes against ischemia/reperfusion injury. However, only conventional‐IPC reduced the Ca^2+^‐loading during metabolic inhibition and this was independent of any change in *sarc*K_ATP_ channel activity but was associated with a reduction in Na^+^‐loading, reflecting a decrease in Na/H exchanger activity. Remote‐IPC delayed opening of the MPT pores in response to ROS, which was dependent on PKC*ε* and NOS‐signaling. These data show that remote‐IPC inhibits MPT pore opening to a similar degree as conventional IPC, however, the contribution of MPT pore inhibition to protection against reperfusion injury is independent of Ca^2+^‐loading in remote IPC. We suggest that inhibition of the MPT pore and not Ca^2+^‐loading is the common link in cardioprotection between conventional and remote IPC.

## Abstract (precis)

Remote ischemic preconditioning (IPC) provides a similar level of protection against ischemia–reperfusion injury to that of conventional‐IPC. This study shows that unlike conventional‐IPC, this was independent of any reduction in Na or Ca^2+^‐loading during the simulated ischemic event but results from a direct PKCε‐dependent inhibition of the mitochondrial permeability transition pore.

## Introduction

Reperfusion of ischemic myocardium induces substantial cellular injury resulting from mechanical and biochemical necrotic injury (Honda et al. 1047; Piper et al. 2004; Halestrap 2006), which involves substantial Ca^2+^‐loading during ischemia, driven by the “coupled exchanger” mechanism between the sodium/hydrogen exchanger (NHE) and sodium/calcium exchanger (NCX) (Tani and Neely 1989; Allen and Xiao 2003). Early in reperfusion, the mitochondria become reenergized and the membrane potential repolarizes, leading to the production of ATP and ROS (Rodrigo and Standen 2005a; Garcia‐Dorado et al. 2012). This then combines with the high [Ca^2+^]_i_ to trigger large SR‐driven Ca^2+^‐oscillations resulting in strong hypercontracture inducing mechanical injury (Inserte et al. 2002; Kevin et al. 2003; Piper et al. 2004; Rodrigo and Standen 2005a) and opening of the mitochondrial permeability transition (MPT) pore resulting in biochemical driven necrosis (Griffiths and Halestrap 1995; Hausenloy et al. 2004).

Ischemic preconditioning (IPC), in which the heart is subject to brief periods of ischemia (~5 min) interspersed with reperfusion, renders the myocardium resistant to reperfusion injury (Murry et al. 1986; Yellon and Downey 2003). The cellular mechanisms of this cardioprotection have been studied in depth, and as expected IPC has been shown to reduce cytosolic Ca^2+^‐loading and SR‐driven Ca^2+^‐oscillations and therefore hypercontracture‐induced mechanical injury (Garcia‐Dorado et al. 2006; Rodrigo and Samani 2008) and MPT pore opening resulting in biochemical injury (Javadov et al. 2003; Argaud et al. 2004; Garcia‐Dorado et al. 2006), although mechanical damage in the form of reperfusion rigor like contractures independent of Ca^2+^‐loading may also be targeted (Abdallah et al. 2010). Increased Ca^2+^‐loading is a common denominator in both reperfusion hypercontracture and MPT pore formation (Garcia‐Dorado et al. 2012) and it is not surprising therefore, that improved Ca^2+^‐regulation is also seen following IPC (Ylitalo et al. 2000; Rodrigo and Samani 2008; Waldenstrom et al. 2012).

Remote ischemic preconditioning (rIPC) was first described by Przyklenk et al. (1993), who found that a conditioning stimulus applied to the circumflex coronary artery protected the “remote” myocardium supplied by the left anterior descending artery, leading the authors to suggest that the ischemia in one vascular bed resulted in the release of a cardioprotective factor that travelled to the neighboring tissue. Since then, many studies have extended this initial observation, to show that the conditioned muscle bed releases signaling molecules (Shimizu et al. 2009), which then travels to the myocardium in the blood and through the activation of GPCRs (Surendra et al. 2013) triggers a signaling cascade that confers the protective phenotype (Hausenloy and Yellon 2008). However, although rIPC is thought to involve similar humoral signaling agents to the conventional IPC, the cellular mechanisms involved in rIPC cardioprotection are less well defined than for conventional IPC. In particular, while conventional IPC reduces Ca^2+^‐loading (Garcia‐Dorado et al. 2006; Rodrigo and Samani 2008) and inhibits MPT pore opening (Hausenloy et al. 2002) involving an increase in the threshold to Ca^2+^‐mediated MPT pore opening (Argaud et al. 2004), the impact of rIPC on Ca^2+^‐loading and whether this is directly involved in preservation of mitochondrial function and inhibition of the MPT pore is not clear (Hausenloy and Yellon 2008). Indeed, while rIPC by transient hindlimb ischemia of rabbits preserves mitochondrial structure and function, this is not as a result of a direct reduction in sensitivity of the MPT pore to Ca^2+^‐induced opening and may involve *mito*K_ATP_ channel activation in some way (Wang et al. 2008).

We have previously shown that myocytes isolated by enzymatic digestion of intact hearts subject to conventional IPC (3 cycles), were protected against metabolic inhibition and reenergization‐induced loss of contractile function and Ca^2+^‐homeostasis, and that although part of this protection resulted from the reduction in Ca^2+^‐loading during metabolic inhibition, an addition mechanism involving the MPT pore may also be triggered (Rodrigo and Samani 2008).

We have therefore set out to look directly at the impact of rIPC on Ca^2+^‐regulation and cardioprotection in comparison to conventional IPC. More specifically at the role of the resting membrane potential (*sarc*K_ATP_ channel activation), the “coupled exchanger” mechanism and MPT pore formation in this protection, using electrophysiology to measure *sarc*K_ATP_ channel activity and fluorescence microscopy to measure diastolic resting membrane potential (RMP) of the cell and mitochondrial membrane potential (Δ*ψ*m) as a surrogate marker of MPT pore opening (Hausenloy et al. 2004), and intracellular Ca^2+^, Na^+^, and pH. To facilitate this, we have used a cellular model of rIPC in which the effluent collected during the reperfusion phase of IPC cycles of isolated rat hearts, is collected and used to remotely condition naïve myocytes isolated from control hearts, thus replicating the method adopted by Dickinson et al., who first showed that the effluent from preconditioned rabbit hearts was able to reduce infarct size in intact whole naïve hearts (Dickson et al. 1999). This model of rIPC was then compared to our model of conventional IPC, in which myocytes are isolated from whole hearts subject to three cycles of IPC (Rodrigo and Samani 2008).

## Materials and Methods

### Isolation of adult rat ventricular myocytes and conditioning protocols

Adult male Wistar rats (2050–350 g) were killed by cervical dislocation, and the heart removed rapidly and immersed in cold Tyrode solution. The hearts were perfused using a constant flow (peristaltic pump) Langendorff apparatus and single ventricular myocytes were isolated by enzymatic digestion of control “naïve” rat hearts, as previously described (Rodrigo and Samani 2008). To obtain IPC‐myocytes, hearts were preconditioned by three cycles of 5‐min global ischemia induced by switching off the peristaltic pump, and 5‐min reperfusion, followed by enzymatic digestion of the heart (Rodrigo and Samani 2008). In addition, 3 mL of effluent was collected at the start of the reperfusion periods of the preconditioning cycles. This “conditioned perfusate” was then frozen and stored at −20°C until used within 8 weeks. To simulate rIPC, 1 mL of naïve myocyte suspension isolated from normal rat hearts, was treated with 1 mL of the conditioned perfusate for 15 min at 35°C. This investigation complied with the university's animal care and welfare guidelines, which conforms to the UK Animals (Scientific Procedures) Act, 1986 and the Guide for the Care and Use of Laboratory Animals published by the US National Institutes of Health (NIH Publication No. 85‐23, revised 1996).

### Ischemic pelleting

Myocytes were centrifuged to form a dense pellet of cells, the supernatant was removed to leave a thin layer of solution above the pellet and a layer of mineral oil (250 *μ*L) added to prevent gas exchange and myocytes were incubated at 35°C for 30 min to simulate ischemia. Reperfusion was achieved by sampling (50 *μ*L) of cells through the oil and triturating the myocytes in oxygenated normal Tyrode with a 1‐mL plastic pipette to simulate mechanical injury (30s) and incubating at 35°C for a further 10 min, before staining with calcein and propidium iodide to detect viable and necrotic myocytes (adapted from (Ganote 1983)). Viable and necrotic cells were then identified by fluorescence microscopy of four random fields of cells (>100 cells/field).

### Metabolic inhibition and reenergization

Ischemia was simulated by superfusion of electrically stimulated myocytes with metabolic inhibition (MI) Tyrode for 10 min, which contained 2 mmol/L NaCN and 1 mmol/L iodoacetic acid in substrate‐free Tyrode, followed by superfusion of normal Tyrode for 10 min to reenergize the mitochondria and simulate reperfusion‐injury (Rodrigo and Samani 2008).

### Measurement of *sarc*K_ATP_ channel current using whole‐cell patch clamp

Myocytes were voltage‐clamped in the whole‐cell configuration at a holding potential of −50 mV and stepped to a test potential of 0 mV for 100 ms at 0.1 Hz. Patch pipettes (2–5 MΩ) were filled with a solution containing (in mmol/L) 140 KCl, 5 EGTA, 0.4 ATP, 0.1 GTP, and 10 HEPES, pH7.3. Data were sampled at 5 kHz using PClamp 10 (Axon instruments), filtered at 2 kHz using an Axopatch 200B patch‐clamp amplifier and Digidata 1440A (Axon Instruments). Mean steady‐state current was measured during the final 10 ms of the test pulse and normalized to cell capacitance (pA/pF). *Sarc*K_ATP_ currents were activated by perfusing the myocyte with MI‐Tyrode and the *sarc*K_ATP_ current identified by the addition of glybenclamide (10 *μ*mol/L).

### Fluorescence measurement of intracellular calcium, sodium, and pH and resting membrane potential

Myocytes were loaded with Fura‐2 AM (5 *μ*mol/L) to measure [Ca^2+^]_i_; with SBFI (5 *μ*mol/L) to measure [Na^+^]_i_, and BCECF to measure pH, for 20 min then washed twice with normal Tyrode to remove any extracellular dye. Myocytes were then transferred to a perfusion chamber mounted on the stage of a Nikon inverting microscope (Nikon TE‐2000E) and continuously superfused at 35°C.

To record the resting membrane potential (RMP), myocytes were stained with the voltage sensitive dye bis‐(1,3‐dibutylbarbituric acid) trimethine oxonol (DiBac_4_(3)), that partitions across the sarcolemmal membrane and changes its fluorescence intensity dependent on the membrane potential (Epps et al. 1994; Baczko et al. 2004). Myocytes were treated with 1 *μ*mol/L DiBac_4_(3) for 20 min at room temperature, transferred to the perfusion chamber and constantly superfused with solutions containing 1 *μ*mol/L DiBac_4_(3) at 35°C during experiments. To calibrate DiBac_4_(3) fluorescence signal to resting RMP in mV, fluorescence was recorded in response to changes in the bathing potassium concentration from 2.5, 6, 10 to 20 mmol/L and plotted as F_1_/F_0_. In separate experiments, the resting membrane potential was recorded in isolated control myocytes and superfused with Tyrode with the same range of potassium concentrations to construct a calibration curve from which recordings of DiBac_4_(3) were converted to RMP.

#### Rapid fluorescence measurements

We used a photomultiplier‐based system to make rapid measurements of pH, [Ca^2+^]_i_, and [Na^+^]_i_ from single myocytes. BCECF loaded myocytes were illuminated alternately with 440/490 nm (50 Hz), Fura‐2 loaded myocytes with 340/380 at 50 Hz, and SBFI‐loaded myocytes at 340/380 (1 Hz), using a monochromator and emitted light collected using a photomultiplier tube (Photon Technology International, Horiba Scientific, NJ).

#### Fluorescence imaging from multiple cells

We used an imaging‐based system to make measurements of [Ca^2+^]_i_ (Fura‐2), RMP (DiBac_4_(3)), and mitochondrial membrane potential (TMRE), simultaneously from a number of cells in a single field of view containing 6–10 cells for Fura‐2 or DiBac_4_(3) with a x20 objective, and 2–3 cells for TMRE with a x40 objective using a video‐imaging system (Perkin‐Elmer). Fura‐2 was excited alternately at 340/380 nm using a Lamda DG‐4 rapid system (Sutter Instrument Company, Novato, CA), and the ratio of the emitted light measured (>510 nmol/L) was recorded. Fluorescence cell images were captured every 10s with an ORCA‐ER CCD camera (Hamamatsu) and Volocity 6.1 software (PerkinElmer, Coventry, UK). DiBac_4_(3) was excited at 480 nm and the emitted light intensity at 535 nm with fluorescence images was captured every 30 sec. DiBac_4_(3) fluorescence intensity was normalized to the fluorescence at rest in myocytes superfused with normal Tyrode (*F*_1_/*F*_0_) and converted to RMP in mV using a calibration curve.

Myocytes were electrically field‐stimulated at 1 Hz using platinum electrodes and superfused with Tyrode continuously at 35°C in all fluorescence experiments, except during the NH_4_Cl‐prepulse experiments, when the stimulator was switched off.

#### SBFI measurement of intracellular sodium during metabolic inhibition

**Figure 1.**
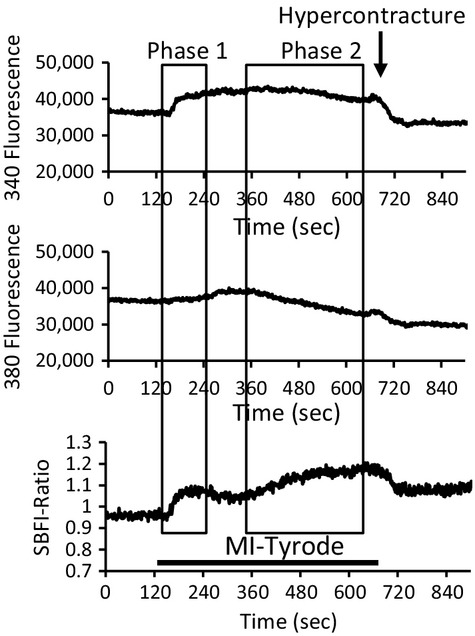
The SBFI fluorescence measurement of intracellular sodium. Record of SBFI‐fluorescence record at *F*_(340)_ and *F*_(380)_ and the ratio *F*_(340/380)_, during perfusion of a control myocyte with MI‐Tyrode.

It has been shown previously that the fluorescence signal of SBFI (*F*_(340)_ and *F*_(380)_) is sensitive to changes in NADH during metabolic inhibition with cyanide (Donoso et al. 1992). This study also showed that the in vivo fluorescence response of SBFI was different to the in vitro characteristics, with the *F*_(380)_ signal showing a decrease in intensity in response to an increase in [Na^+^]_i_ and *F*_(340)_‐signal not responding to changes in [Na^+^]_i_. Figure 1 shows the SBFI‐fluorescence record at *F*_(340)_ and *F*_(380)_ and the ratio *F*_(340/380)_, in which a rapid increase in the *F*_(340)_ fluorescence is detected in MI‐Tyrode, as reported previously (Donoso et al. 1992). However, this rapid increase was absent in *F*_(380)_ signal, which did show a gradual decrease as expected for an increase in [Na^+^]_i_. The ratio *F*_(340/380)_, indicates two phases a rapid increase, which was due to the large increase in F_(340)_ responding to the increase in [NADH], and the gradual increase reflecting an increase in [Na^+^]_i_ which is in agreement with the previous report (Donoso et al. 1992).

### Measurement of time to MPTP opening in intact cells

We used a technique previously described by Hausenloy et al. (2004) to follow opening of the mitochondrial permeability transition pore (MPT pore) in isolated myocytes. Briefly, isolated ventricular myocytes were loaded with 2.5 *μ*mol/L TMRE for 20 min at room temperature. Cells were transferred to the tissue dish of the fluorescence Nikon microscope and constantly superfused with Tyrode solution. At the start of the experiment, cells were exposed to constant illumination at 535 nm and emission at >590 nm was measured every 5 sec using the fluorescence imaging system (Perkin‐Elmer), and the time to MPT pore opening indicated by the increase in fluorescence to 90% of maximum. The increase in fluorescence signal was fitted with a sigmoidal curve and the time taken to 90% of maximal intensity recorded.

### Western blot analysis of PKCε translocation

Western blot analysis of PKC*ε* translocation was performed as previously described (Turrell et al. 2011). Briefly, myocytes were centrifuged at 12,000 × g for 60s at room temperature and the pellet resuspended in ice‐cold, nondetergent lysis buffer. Following homogenization, the lysate was centrifuged at 98,000 × g for 30 min at 4°C to separate the cytosolic fraction (supernatant) and the pellet (membrane fraction) solubilized and spun at 12,000 × g for 10 min at 4°C.

### Drugs and experimental solutions

Normal Tyrode solution contained (mmol/L): NaCl 135, KCl 5, NaH_2_PO_4_ 0.33, Na‐pyruvate 5, glucose 10, MgCl_2_ 1, CaCl_2_ 2, HEPES 10, titrated to pH 7.4 with NaOH. Substrate‐free Tyrode (normal Tyrode with sucrose replacing glucose and NaCl replacing Na‐pyruvate).

Fura‐2, BCECF, SBFI, and TMRE (Molecular Probes Inc.) was dissolved in DMSO containing 5% pluronic acid (5 mmol/L). The PKC*ε* inhibitor peptide *ε*V1‐2 were synthesized by Pepceuticals and were a kind gift from Dr RI Norman, Leicester University.

### Statistical analysis

**Figure 2.**
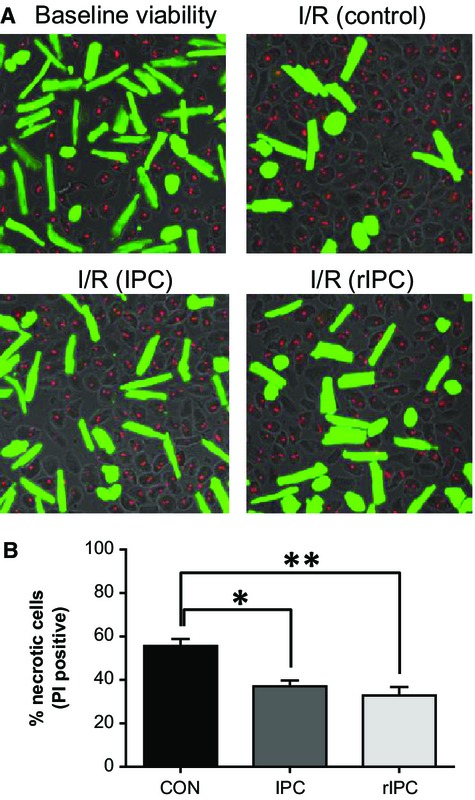
Ischemic preconditioning protects myocytes against simulated ischemia/reperfusion injury. (A) Fluorescent images of isolated ventricular myocyte stained with calcein (green) and propidium iodide (red) to indicate viable and necrotic cells, following 30 min of simulated ischemia and 10 min of reperfusion. (B) Mean data expressed as percentage necrotic cells (PI positive) for control naïve myocytes (*n* = 13; 8, black), conventional IPC myocytes (*n* = 7; 32, dark gray), and remote IPC myocytes (12; 17, light gray). Mean ± SEM; *P < 0.05, **P < 0.01, one‐way ANOVA followed by Tukey's *post hoc* test for significance.

**Figure 3.**
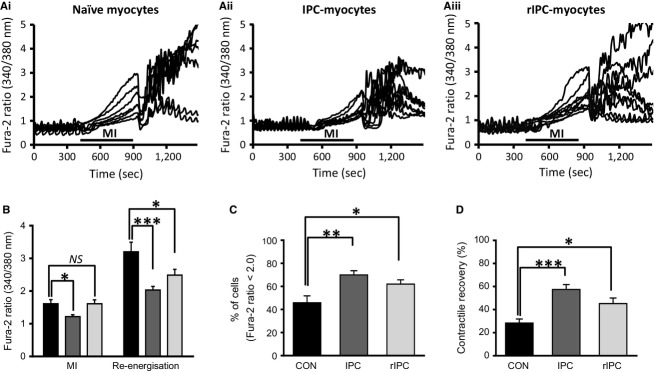
Ca^2+^‐homeostasis in conventional and remotely preconditioned myocytes subject to MI and reenergization. (A) Simultaneous recordings of [Ca^2+^]_i_ from (i) seven control myocytes; (ii) nine conventional IPC‐myocytes, and (iii) nine rIPC‐myocytes from a single field of view during metabolic inhibition (8 min) and reenergization (10 min). (B) The mean Fura 2 ratio at the end of 8 min MI and 10 min reenergization for control naïve myocytes (*black*), conventional IPC‐myocytes (*dark gray*), and remote IPC‐myocytes (*light gray*). (C) Percentage of myocytes with a diastolic Fura‐2 ratio <2.0 after 10 min of reenergization. (D) Percentage of myocytes contracting in response to field stimulation after 10 min of reenergization. Mean ± SEM; *P < 0.05, **P < 0.01, ***P < 0.001, one‐way ANOVA followed by Tukey's post hoc test for significance. Control myocytes = 6 hearts; 23 observations and 185 myocytes, conventional IPC = 6; 28, and 254, remote IPC = 6; 33, and 315.

Data are presented as mean of the experimental observations ± SEM, with the number of hearts and experimental observation indicated as (*n = hearts; experiments*). For calculations of percent necrotic cells (Fig. 2), the mean from four randomly selected fields‐of‐view containing >100 cells per experimental observation counted and the mean of this mean reported. For calculation of Fura‐2 ratio and percentage Fura‐2 ratio <2.0, the mean from a field‐of‐view containing 8–12 cells per experimental observation was calculated and for recovery of contractile function the mean from a field‐of‐view containing 15–20 cells calculated, and the mean of these mean reported (Fig. 3). Statistical significance was determined using a one‐way ANOVA with Tukey's post hoc test using GraphPad Prism5. *P *<**0.05 were considered statistically significant.

## Results

### Remote IPC of naïve myocytes with “conditioned perfusate” protects isolated myocytes against simulated ischemia–reperfusion injury

We have previously shown that “IPC‐myocytes” enzymatically isolated from intact hearts subjected to IPC, were significantly protected against ischemia–reperfusion injury simulated using a cell pelleting technique (Rodrigo and Samani 2008). We looked at the ability of “conditioned perfusate” collected as the effluent from intact hearts subject to three cycles of IPC, to remotely condition and protect “naïve” myocytes (rIPC‐myocytes) against ischemia/reperfusion injury. Figure 2A are representative images of myocytes subject to reperfusion injury in this way and 2B is the average data taken from such experiments and shows that the percentage cell death in control naïve myocytes at 55.6 ± 3.3% (*n* = 8; 16) was decreased in IPC‐myocytes to 37.0 ± 2.8% (*n* = 7; 14, *P* < 0.05) in remote IPC myocytes to 32.9 ± 3.9% (*n* = 8; 15, *P* < 0.01).

We looked at the role of adenosine and opioid receptors, which have been shown to be involved in remote IPC in‐vivo, in our model of rIPC using 8p‐SPT (100 *μ*mol/L) a nonspecific adenosine receptor blocker and naloxone (100 *μ*mol/L) an opioid receptor present at the time of treatment of naïve myocytes with the conditioned perfusate. Either of these agents fully blocked the ability of conditioned perfusate to protect the naïve myocytes against I/R injury from 32.8 ± 3.7% in rIPC‐myocytes (*n* = 7; 13), to 48.3 ± 9.5% with 8p‐SP (*n* = 3; 6, *P* < 0.05) and 54.1 ± 8.1% with naloxone (*n* = 3; 6, *P* < 0.01). The data show that remote IPC of naïve myocytes significantly reduces the level of necrotic injury to similar levels seen in conventional IPC, and this involves adenosine and opioids.

### IPC and remote IPC protect isolated myocytes against Ca^2+^‐overload after metabolic inhibition and reenergization

Ca^2+^‐influx resulting in Ca^2+^‐overload has been implicated in the injury during reperfusion of the ischemic myocardium (Garcia‐Dorado et al. 2012), and Ca^2+^‐loading has been shown to be reduced by IPC in single ventricular myocytes (Rodrigo and Samani 2008). However, the ability of rIPC to alter Ca^2+^‐loading of myocytes is not known. We therefore compared the characteristics of Ca^2+^‐loading during MI and reenergization in control naïve‐myocytes, IPC‐myocytes, and rIPC‐myocytes subject to remote IPC with conditioned perfusate.

Figure 3A are typical records of [Ca^2+^]_i_ from a single field of (i) naïve‐myocytes (ii) IPC myocytes, and (iii) rIPC‐myocyte subjected to metabolic inhibition (8 min) and reenergization (10 min). As the imaging system collects ratio images every 10 sec, this results in aliasing of the fast Ca^2+^‐transients, which now appear as slow rhythmic oscillations as reported previously (Rodrigo and Samani 2008). However, these records show a failure of excitation–contraction coupling indicated by the loss of Ca^2+^‐oscillations during MI followed by a steady increase in [Ca^2+^]_i_ which is reduced in IPC‐myocytes but not in rIPC‐myocytes. Reenergization of the myocytes results in a rapid drop in [Ca^2+^]_i_ in all myocytes (naïve, IPC, and rIPC) before a steady increase (Fura2 ratio >2.0) in five of seven naïve myocytes, whereas only two of nine IPC‐myocytes and three of nine rIPC‐myocytes showed this uncontrolled increase. The mean of these experiments show that the increase in mean diastolic [Ca^2+^]_i_ at the end of MI phase was reduced in IPC but not rIPC‐myocytes. Following 10 min of reenergization the mean diastolic ratio had increased in control naïve myocytes to 3.2 ± 0.2 (*n* = 22) but this was reduced to 2.0 ± 0.1 (*n* = 8; 28) in IPC‐myocytes and to 2.5 ± 0.2 (*n* = 7; 33) in rIPC‐myocytes (Fig. 3B).

It is apparent from the original records in part A that a clear division exists between those myocytes that recover a low diastolic [Ca^2+^]_i_ (Fura2 ratio <2.0) and others where the [Ca^2+^]_i_ increases dramatically. We therefore looked at the percentage of myocytes able to maintain a low level of diastolic [Ca^2+^]_i_ (Fura‐2 ratio), and those that recover contractile function determined as the percentage of myocytes able to contract in response to electrical field stimulation, at the end of 10 min reenergization. Figure 3C shows that percentage of cells able to maintain a low diastolic [Ca^2+^]_i_ (Fura2 ratio <2.0) at the end of 10 min reenergization, is significantly greater in both IPC at to 69.9 ± 3.7% (*P* < 0.01) and rIPC‐myocytes at 61.9 ± 3.7% (*P* < 0.05), compared to naïve‐myocytes at 45.8 ± 5.9%. This relationship is similar to the recovery of contractile function, with the difference between IPC and rIPC also approaching significance of *P* = 0.07 (Fig. 3D).

### IPC and rIPC do not alter the activation of *sarc*K_ATP_ current or the response of the resting membrane potential to metabolic inhibition

The increase in [Ca^2+^]_i_ that result during oxidative stress (ischemia, metabolic inhibition) has been shown to involve reverse‐mode NCX, driven by an accumulation of [Na^+^]_i_ (Tani and Neely 1989; Imahashi et al. 2005) and membrane depolarization (Baczko et al. 2003). It is suggested that this Ca^2+^ accumulation, is reduced by the opening of *sarc*K_ATP_ channels which hyperpolarizes the diastolic RMP (Baczko et al. 2004). We have therefore measured *sarc*K_ATP_ current density and the RMP using the membrane‐potential‐sensitive fluorescent dye DiBac_4_(3) in control naïve‐myocytes and compared this to IPC‐myocytes and rIPC‐myocytes, during superfusion with MI‐Tyrode.

**Figure 4.**
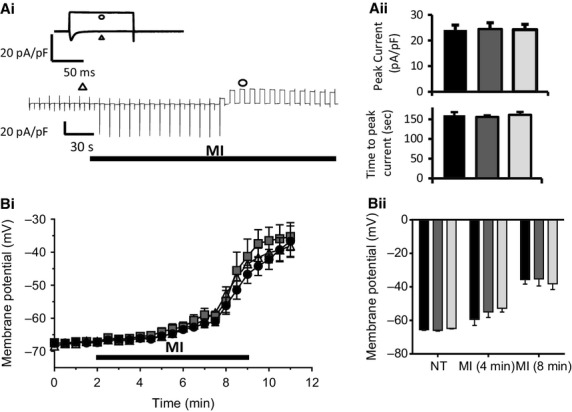
Resting membrane potential in conventional and remotely preconditioned myocytes subject to MI and reenergization. (A) (i) Concatenated record of whole‐cell membrane currents from a control myocyte showing the activation of *sarc*K_ATP_ current during perfusion with MI‐Tyrode. (Insert) Membrane current in response to depolarization from the holding potential of −50 mV to the test potential of 0 mV, in normal Tyrode (triangle) and at the peak *sarc*K_ATP_ current amplitude (circle). (ii) Bar chart showing the peak *sarc*K_ATP_ current density (top) and time to peak current during metabolic inhibition in control (black), conventional IPC (dark gray), and remote IPC myocytes (light gray). (B) (i) Record of resting membrane potential measured using DiBac_4_(3) fluorescence in control, conventional IPC, and remote IPC myocytes. Values are the mean ± SEM from a single experimental run where the RMP was determined simultaneously from 5 to 8 myocytes in a single field of view. (ii) Mean data ± SEM of the resting membrane potential recorded in normal Tyrode and in MI‐Tyrode at 4 and 8 min, from control naïve (black), conventional IPC (dark gray), and remote IPC myocytes (light gray). ANOVA followed by Tukey's post hoc test for significance. Control naïve‐myocytes = 6 hearts; 62 cells, conventional IPC‐myocytes = 4; 26, remote IPC‐myocytes = 6; 63.

The data in Figure 4A show no effect of conventional IPC or rIPC on the peak *sarc*K_ATP_ current during metabolic inhibition. Fluorescence measurements show no difference in the basal RMP recorded from control myocytes −65.5 ± 3.1 mV, IPC‐myocytes −65.9 ± 2.7 mV, and rIPC myocyte −64.8 ± 2.1 mV. The induction of metabolic inhibition results in a steady depolarization of the RMP in IPC‐myocytes to −35.3 ± 4.1 mV and naïve rIPC myocyte −38.1 ± 3.4 mV after 8 min superfusion with MI‐Tyrode, which was not significantly different to control myocytes at −35.6 ± 2.7 mV (Fig. 4B). We have previously shown the conditioning of myocytes with pharmacological conditioning drug diazoxide, caused a delay in action potential failure during metabolic inhibition, indicating that diazoxide‐treatment delayed the onset to *sarc*K_ATP_ activation (Rodrigo et al. 2004). However, both IPC and rIPC had no significant effect on the time‐course of the activation of *sarc*K_ATP_ current or to depolarization in MI‐Tyrode.

### IPC but not rIPC reduces Na‐loading during metabolic inhibition

If the increase in [Ca^2+^]_i_ is due to reverse‐mode NCX activity and there is no significant effect of IPC or rIPC on diastolic RMP during MI, this could result from a difference in Na‐loading. We therefore measured SBFI‐ratio, as an indicator of [Na^+^]_i_, in control naïve‐myocytes, IPC‐myocytes, and rIPC‐myocytes during MI.

**Figure 5.**
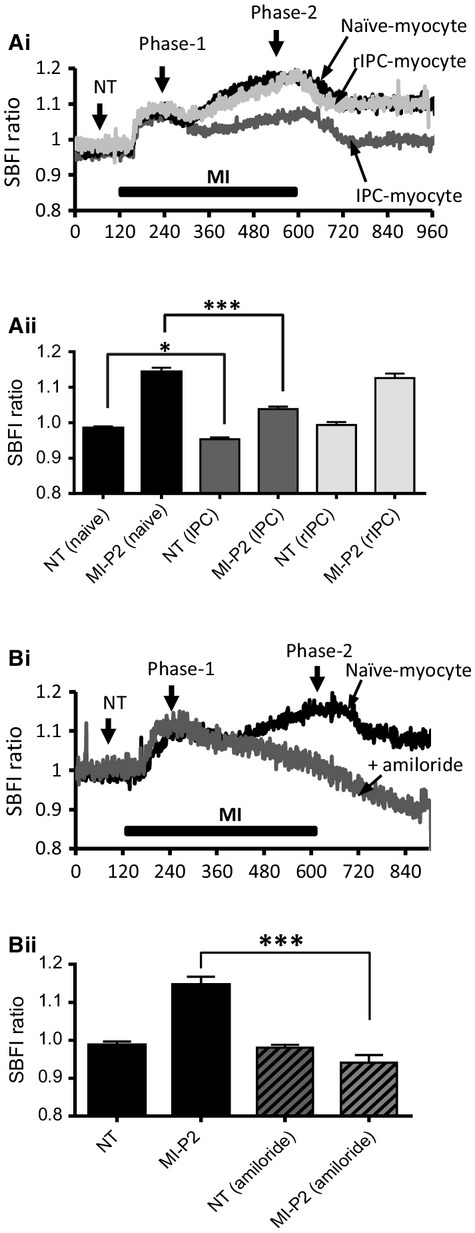
Intracellular sodium concentrations (SBFI ratio) in conventional and remotely preconditioned myocytes subject to MI and reenergization. (A) (i) Recordings of SBFI ratio as an indicator of [Na^+^]_i_ in a control naïve‐myocyte (black trace), conventional IPC‐myocyte (dark gray), and remote IPC myocyte (light gray) during perfusion with MI‐Tyrode. (ii) Mean data ± SEM of the SBFI ratio recorded in normal Tyrode (NT) and at the end of 8 min perfusion with MI‐Tyrode (as indicated on 4Ai). (B) (i) Recordings of SBFI ratio in a control naïve‐myocyte (black trace) and a naïve‐myocyte in the presence of amiloride (dark gray) during perfusion with MI‐Tyrode. (ii) Mean data ± SEM of the SBFI ratio recorded in normal Tyrode (NT) and at the end of 8 min perfusion with MI‐Tyrode. *P < 0.05, ***P < 0.001, one‐way ANOVA followed by Tukey's post hoc test for significance. Control naïve‐myocytes = 5 hearts; 50 cells, conventional IPC‐myocytes = 5; 45, remote IPC‐myocytes = 5; 38, and Control naïve‐myocytes + amiloride = 3; 18.

Figure 5Ai shows records of SBFI fluorescence ratio from a control naive, conventional IPC, and rIPC‐myocyte. Superfusion with MI‐Tyrode resulted in a biphasic increase in the ratio of SBFI fluorescence in control myocytes, with an initial rapid increase (Phase1) followed by a slower steady increase with continued superfusion with MI‐Tyrode (Phase 2). Phase 1 characterized by this rapid increase in SBFI‐ratio was similar in all three myocyte types and is probably due to the increase in NADH fluorescence associated with inhibition of electron transport by cyanide (Donoso et al. 1992). However, phase 2 of the increase in SBFI‐ratio reflects the increase in [Na^+^]_i_ as shown by the decrease in F_380_, which has been shown to decrease fluorescence in response to an increase in [Na^+^]_i_ ((Donoso et al. 1992) and Fig. 1).

Figure 5Aii, is a bar chart of the mean data from such experiments and shows a significantly lower basal SBFI‐ratio in IPC‐myocytes compared to control naïve‐myocytes at 0.95 ± 0.005 (*n* = 50; 5) versus 0.98 ± 0.005 (*n* = 5; 45, *P* < 0.01) recorded in normal Tyrode. In addition, the increase in SBFI‐ratio at the end of 8 min superfusion with MI‐Tyrode was significantly lower in IPC‐myocytes at 1.04 ± 0.006 versus 1.14 ± 0.1 in control naïve‐myocytes (*P* < 0.0001) at the end of MI‐Tyrode (8 min). In contrast, the SBFI‐ratio at rest and during MI was not significantly different in rIPC‐myocytes (*n* = 8; 38) compared to control naïve myocytes.

Figure 5Bi is a record of SBFI fluorescence ratio from a naïve‐myocyte compared with the response of a naïve‐myocyte in the presence of amiloride (5‐(*N*,*N*‐Hexamethylene)amiloride), which completely abolished phase 2 of the increase in SBFI ratio, which reflects the increase in [Na^+^]_I_ but had no effect on phase‐1 which is driven by changes in NADH. The bar chart of the mean data (Fig. 5Bii) shows that inhibition of the NHE prevents the MI‐induced increase in [Na^+^]_i_.

### IPC reduces rate of recovery from acidosis

Inhibition of the NHE has been shown to reduce Na‐loading that occurs during ischemia (Williams et al. 2007) or metabolic inhibition of isolated myocytes ((Baartscheer et al. 2011) and Figure 4B). Ischemic preconditioning of an isolated rat heart reduced Na‐loading during prolonged ischemia, which reflected a reduction in activity of the NHE (Xiao and Allen 1999). We therefore compared the activity of the NHE in control, IPC‐myocytes, and naïve myocytes subject to remote IPC with conditioned perfusate, by recording the rate of recovery from an acid‐load in response to an NH_4_Cl‐prepulse protocol (20 mmol/L for 5 min).

**Figure 6.**
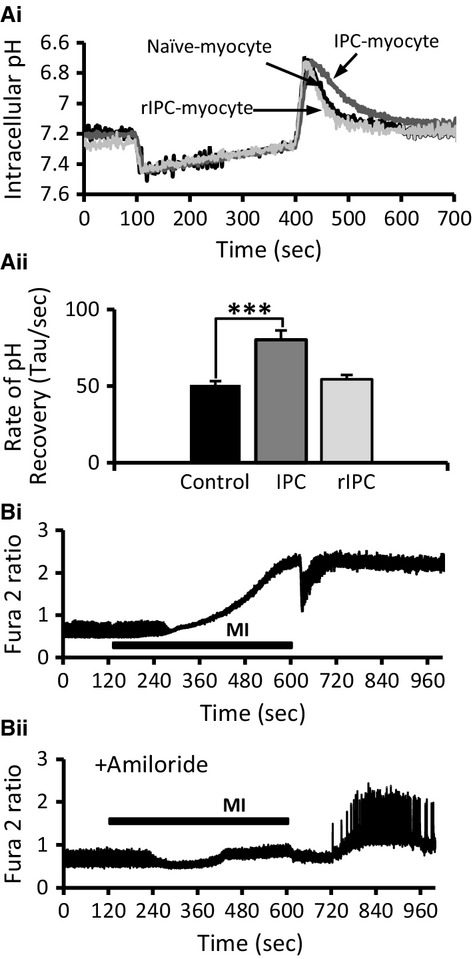
Intracellular pH in conventional and remotely preconditioned myocytes in response to an NH_4_Cl acid‐pulse (A) (i) Record of intracellular pH from a control naïve‐myocyte (black trace), conventional IPC‐myocyte (dark gray trace), and remote IPC myocyte (light gray), in response to 20 mmol/L NH_4_Cl for 5 min. Mean data ± SEM of the rat of recovery of intracellular pH from the acid load induced by the NH_4_Cl pulse in control naïve myocytes (black), conventional IPC myocytes (dark gray), and remotely conditioned myocytes (light gray). ***P < 0.001, one‐way ANOVA followed by Tukey's post hoc test for significance. Control naïve myocytes = 3 hearts; 16 cells, conventional IPC myocytes = 3; 25, remote IPC = 3; 21. (B) (i). Representative record of Fura‐2 ratio from a single control myocytes during MI and reenergization, and (ii). In the presence of amiloride. This result was seen in seven cells from three different hearts.

The resting intracellular pH was not affected by IPC or rIPC with a pH of 7.22 ± 0.02 (*n* = 3; 16) in control myocytes; 7.17 ± 0.02 (*n* = 3; 25) in IPC‐myocytes, and 7.21 ± 0.05 (*n* = 3; 21) in rIPC‐myocytes. Addition of NH_4_Cl (20 mmol/L) to the superfusate caused in a rapid intracellular alkalinization, which on washout of NH_4_Cl resulted a rapid acidification and the peak acidification was similar in all three myocyte groups (Figure 6A). However, the rate of recovery from the acid load, as determined from the exponential time constant for the recovery, was significantly reduced to 80.1 ± 6.2 sec in IPC‐myocytes from 50.7 ± 2.5 sec in control myocytes (*P* < 0.001). The exponential time constant was not altered in rIPC‐myocytes at 54.4 ± 2.9 sec (NS).

Figure 6 Bi shows a record (photomultiplier system) of fura‐2 ratio from a single control myocytes during MI and reenergization, and reveals the loss of Ca^2+^‐transients during MI and the subsequent rise in Fura‐2 ratio. In the presence of amiloride, this increase in Fura‐2 ratio is absent during MI (Figure 6Bii). This result was seen in seven cells isolated from three separate hearts.

### The role of PKCε signaling in cardioprotection

**Figure 7.**
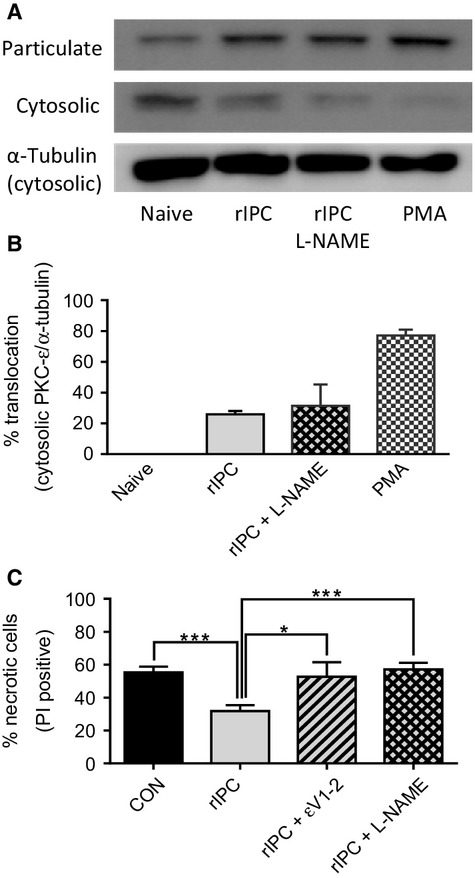
Remote ischemic preconditioning causes translocation of PKC*ε* (A) A representative western blot of protein extracted from naïve myocytes, rIPC myocytes, and rIPC myocytes + L‐NAME (L‐NAME present during the remote conditioning phase) and PMA treated naïve‐myocytes. (B) Bar chart of the percentage translocation of PKC*ε*, calculated from the disappearance of PKC*ε* from the cytosol, when compared with naïve‐myocytes, from control naïve myocytes (black); rIPC myocytes (light gray); rIPC myocytes conditioned in the presence of L‐NAME (pale‐gray diamonds) and myocytes treated with PMA (black/white checks). The cytosolic fraction contained the fraction of PKC*ε* that is not translocated to the particulate (membrane) fraction and is normalized to *α*‐tubulin present in the cytosolic fraction. (C) Bar chart of percentage necrotic cells (PI positive) for control naïve myocytes (black); rIPC myocytes (light‐gray); rIPC conditioned in the presence of PKC*ε*V1‐2 (pale‐gray hashed) or L‐NAME (pale‐gray diamonds). Mean ± SEM; *P < 0.05, **P < 0.01, one‐way ANOVA followed by Tukey's post hoc test for significance.

Involvement of PKC*ε* signaling in conventional and remote IPC is well documented (Wolfrum et al. 2002; Inagaki et al. 2006). We determined to look at the role of PKC*ε* in our rIPC myocytes as further support to the validity of our model for remote IPC. Myocytes were isolated from hearts subject to conventional IPC (three cycles of 5 min ischemia and 5 min of reperfusion). Naïve myocytes were isolated from control hearts and these were either remotely conditioned for 15 min to produce rIPC‐myocytes or treated with PMA (1 *μ*mol/L) as a positive control. Western blot analysis of PKC*ε* shows significant translocation from the cytosolic fraction to the particulate fraction in rIPC myocytes comparable to that in conventional IPC myocytes (Figure 7A). The data were quantified using densitometry readings of PKC*ε* bands and calculating the disappearance of PKCe from the cytosolic fraction (naïve‐treated), which was then expressed as a percentage on the naïve band (Figure 7B).

To determine the involvement of PKC*ε* in cardioprotection, naïve myocytes were incubated with the Tat‐PKC*ε*V1‐2 inhibitor peptide conjugate (100 nmol/L) (Turrell et al. 2011), and the involvement of NOS through inhibition with L‐NAME (100 *μ*mol/L), prior to being remote IPC with conditioned perfusate. The data show that PKC*ε* or NOS inhibition blocked protection by remote‐IPC of naïve myocytes (Figure 7C) but that NOS inhibition did not prevent PKC*ε* translocation suggesting its involvement downstream of PKC*ε*.

### Both IPC and rIPC delays opening of the mitochondrial permeability transition pore in isolated myocytes

**Figure 8.**
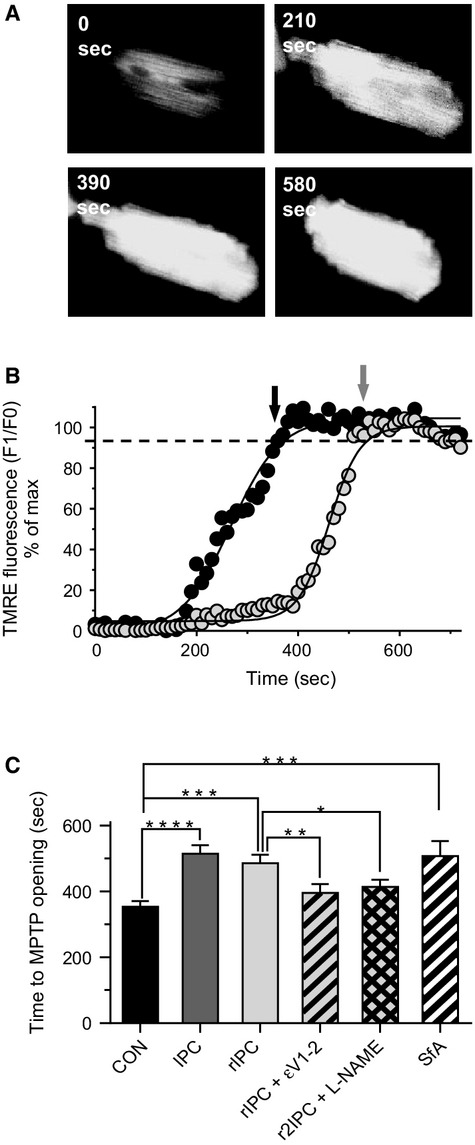
The TMRE fluorescence recorded from conventional and remotely preconditioned myocytes as an indicator of mitochondrial transition pore opening. (A) Fluorescent image of a control myocyte continuously illuminated, illustrating the photodamage due to continuous illumination of TMRE loaded myocyte, at time 0 sec, 210 sec, 390 sec, and 580 sec of illumination. The large increase in fluorescence is visible at 390 sec and at 580 sec the myocyte can be seen to develop a rigor contracture. (B) Trace of TMRE fluorescence from a control myocyte (black circles) and remote IPC myocyte (light‐gray circles). Data points were fitted with a sigmoidal curve and time to increase in fluorescence to 90% of max determined. (C) Mean data ± SEM of the time to 90% increase in fluorescence from control naïve‐myocytes (black), conventional IPC‐myocytes (dark gray), rIPC myocytes (light gray), and control myocytes in the presence of Sanglifehrin‐A (0.5 *μ*mol/L). **P < 0.01, ***P < 0.001, one‐way ANOVA followed by Tukey's post hoc test for significance. Control naïve myocytes = 4 hearts; 22 observations, conventional IPC myocytes = 4; 15, remote IPC = 4; 18, and control + Sanglifehrin‐A = 4; 14.

We have previously shown that additional mechanisms over and above that of Ca^2+^‐loading during MI, are important in the recovery of IPC‐myocytes during reenergization (Rodrigo and Samani 2008) and that inhibition of the MPT pore protects naïve‐myocytes against loss of function and Ca^2+^‐homeostasis following MI and reenergization (Rodrigo and Standen 2005a). The use of TMRE to measure mitochondrial membrane potential has been adopted as an indirect marker of MPT‐pore opening (Hausenloy et al. 2004). However, as MI results in depolarization of the mitochondrial membrane potential due to the inhibition of the electron transport chain (Lawrence et al. 2001), we were not able use this technique to look at opening of MPT pore in response to MI. We therefore looked at the ability of both IPC and rIPC of naïve myocytes to delay the opening of MPT pore in response to stress induced by illumination of myocytes loaded with TMRE (Hausenloy et al. 2004). MPT pore opening was taken as the time to increase in TMRE fluorescence to 90% of maximum (Fig. 8A and B, see methods).

Our data show that opening of MPT pores was significantly delayed from 353 ± 18 sec (22; 4) in control naïve‐myocytes, to 514 ± 26 sec (15; 4, *P* < 0.001) in IPC‐myocytes, and 480 ± 28 sec (18; 4, *P* < 0.01) in rIPC myocytes. This delay in MPT pore opening was similar to that produced by Sanglifehrin‐A (Fig. 8C) and in rIPC‐myocytes, was reversed by the presence of Tat‐PKC*ε*V1‐2 inhibitor peptide conjugate (100 nmol/L) and L‐NAME (100 *μ*mol/L) during treatment with conditioned perfusate.

## Discussion

We have previously shown that ischemic preconditioning of the intact rat heart confers a cardioprotective phenotype to the isolated single ventricular myocytes, and this protection mirrors that seen in the intact heart, which may be linked to a decrease in Ca^2+^‐loading (Rodrigo and Samani 2008). In this study, we demonstrate that the perfusate collected from hearts during bouts of IPC, when applied to naïve myocytes, is able to induce remote IPC and protects isolated ventricular myocytes against simulated ischemia/reperfusion injury. The reduction in Ca^2+^‐loading during metabolic inhibition of IPC‐myocytes reflects a decrease in Na‐loading possibly due to a reduction in activity of the NHE. However, this reduction in Na^+^ and Ca^2+^‐loading during metabolic inhibition is not seen in remote IPC myocytes, even though they are protected against simulated ischemia/reperfusion injury (cell pelleting). The common cellular change in conventional IPC and rIPC‐myocytes, which might explain the cardioprotection in spite of the lack of an effect of Ca^2+^‐loading in rIPC, is the protection against ROS‐induced opening of the MPT pore.

### The relationship between intracellular Na^+^ and Ca^2+^ during metabolic inhibition in conventional and remote IPC

We have shown that IPC of an intact heart prior to enzymatic isolation of single ventricular myocytes, confers protection against ischemia/reperfusion simulated using either a cell pelleting model or metabolic inhibition and reenergization, the latter of which is also associated with a reduction in Ca^2+^‐loading (Shimizu et al. 2009). As the impact of Ca^2+^‐loading on ischemia and reperfusion injury is now well documented (Piper et al. 2004; Halestrap 2006; Garcia‐Dorado et al. 2012), it is possible that this reduction in Ca^2+^‐loading might contribute to the cardioprotection we observe, and an understanding of the mechanism for this reduction in Ca^2+^‐loading is important.

Since their discovery by Noma in 1984 (Noma 1983), the *sarc*K_ATP_ channels have been suggested to play a cardioprotective role (Rodrigo and Standen 2005b) and while many reports have suggested a role for the channels as an end‐effector for IPC this has been contested in favor of the mitochondrial *mito*K_ATP_ channel (Garlid et al. 2003; Gross and Peart 2003; Rodrigo and Standen 2005b). Activation of the *sarc*K_ATP_ channels is suggested to reduce Ca^2+^‐loading through a reduction in action potential duration (Lederer et al. 1989) and clamping of the diastolic membrane potential negative to the reversal potential of the NCX (Baczko et al. 2003, 2004). The use of isolated cells in our study has facilitated direct electrophysiological rather than pharmacological studies, which are often criticized on the basis of selectivity of the drugs that target *sarc*K_ATP_ channels (Rainbow et al. 2005; Rodrigo and Standen 2005b). We did not find any difference in either the amplitude of the *sarc*K_ATP_ current or in its time to activation during metabolic inhibition in conventional IPC or rIPC myocytes. Neither was there any significant effect on the diastolic membrane potential during metabolic inhibition or in the time to depolarization to −45 mV, the calculated NCX reversal potential at rest (Baczko et al. 2003), showing no direct effect of *sarc*K_ATP_ channel activity on Ca^2+^‐loading secondary to depolarization of the RMP. However, it is possible that an involvement of the *sarc*K_ATP_ channel in the control of energy generation, transfer, and utilization systems may occur (Crawford et al. 2002; Gumina et al. 2003).

The role of the coupled exchanger mechanism (NHE and NCX) in which the NHE drives the accumulation of [Na^+^]_i_ coupled to the persistent Na‐channels, and the NCX the increase in [Ca^2+^]_i_ during ischemia is now well established (Tani and Neely 1989; Ju et al. 1996; Garcia‐Dorado et al. 2012), and inhibitors of either the NHE and NCX have been shown to reduce reperfusion injury (Karmazyn 1999; Inserte et al. 2002; Imahashi et al. 2005). We have found a similar coupling of Na^+^‐loading to Ca^2+^‐loading in isolated myocytes during metabolic inhibition, and that both the increase in [Na^+^]_i_ and [Ca^2+^]_i_ is blocked by amiloride, which shows the direct involvement of the NHE in Na^+^ and Ca^2+^‐loading during metabolic inhibition (Figs. 5 and 6). Due to its central role in ischemia/reperfusion injury, the NHE has previously been suggested as an end‐effector of IPC (Gumina et al. 2001; Yellon and Downey 2003), even though the conditions resulting from ischemia/reperfusion, seem likely to stimulate NHE activity (Kandasamy et al. 1995; Haworth et al. 2003). The first experimental evidence to show a link between the NHE and IPC‐protection was presented by Xiao and Allen (1999), who demonstrated that; the protection from IPC and inhibition of the NHE was not additive and the rise in [Na^+^]_i_ during the reperfusion phase was reduced in IPC hearts. However, other studies have suggested that NHE inhibitors work separately to IPC (Shipolini et al. 1997; Gumina et al. 1999), and PKC*ε* activation, which is integral to signaling during IPC, stimulates NHE activity (Kandasamy et al. 1995). Our data show a significant slowing in the rate of recovery of myocytes from an acid‐load induced by NH_4_Cl in IPC‐myocytes, suggesting an inhibition of NHE activity. This inhibition of the NHE activity is not seen in rIPC, which do not show the decreased Na^+^ and Ca^2+^‐loading during metabolic inhibition but paradoxically still exhibit protection against ischemia/reperfusion injury simulated using a cell pelleting technique.

The mechanism by which conventional IPC leads to inhibition of NHE‐1 activity in the isolated cardiomyocytes is not clear, but does not appear to necessarily follow PKC*ε* translocation, as we found similar levels of PKC*ε* translocation in both conventional‐IPC and rIPC‐myocytes where inhibition of NHE activity was not evident (Fig. 7). Conventional ischemic preconditioning involves the release of signaling molecules by the ischemic tissue and remote preconditioning is also thought to involve signaling molecules released from the ischemic tissue and in our model of rIPC, the perfusate which contains these signaling molecules released by the ischemic heart tissue was applied to naïve myocytes and indeed protection was blocked by inhibition of adenosine and opioid receptors. However, the two models have obvious differences that may account for the change in NHE activity and thereby Na^+^ and Ca^2+^‐loading. Remotely conditioned myocytes are not subject to the additional effects of ischemia during the conditioning stimulus namely; anoxia/reoxygenation, acidosis, ROS, and local build of extracellular metabolites. ROS (superoxide and H_2_O_2_) production during the brief episodes of hypoxic preconditioning has been shown (Vanden Hoek et al. 1998) and seems likely to contribute to the signaling process that leads to the cardioprotection of IPC (Yellon and Downey 2003). An interesting study has showed that insulin induced an H_2_O_2_‐dependent decrease in intracellular tyrosine phosphatase activity, which resulted in a decrease in NHE activity in mesenteric arterioles (Boedtkjer and Aalkjaer 2009).

### Is a reduction in Ca^2+^‐loading during ischemia/metabolic inhibition a prerequisite to cardioprotection by remote IPC?

Many studies have now shown the inhibition of the NHE is cardioprotective and this is often linked to a reduction in Ca^2+^‐overload, although cause and effect is not always shown. However, the inability of rIPC to inhibit NHE activity and reduce Ca^2+^‐loading of myocytes, while still protecting against simulated ischemia/reperfusion injury, suggests that Ca^2+^‐loading is not essential for the cardioprotection, at least in the setting of rIPC. The mechanism by which Ca^2+^‐overload injury is thought to lead to reperfusion injury, involves the triggering of mechanical injury through the development of a strong hypercontracture, in which high [Ca^2+^]_i_ combines with the availability of ATP from the reenergized mitochondria and the SR to induce large scale oscillations in [Ca^2+^]_i_ that trigger both hypercontraction and open of the MPT pore (Garcia‐Dorado et al. 2012). We have previously shown that the hypercontracture, which is responsible in part for the contraction band necrosis, is dependent on reenergization of the mitochondria and the availability of ATP through the repolarization of the reenergized mitochondria following metabolic inhibition and not on the rise in [Ca^2+^]_i_ during the MI (Rodrigo and Standen 2005a). We did, however, find that the presence of cyclosporine‐A or Sanglifehrin‐A while having no effect on the development of hypercontracture, reduced the percentage of cells that lost Ca^2+^‐homeostasis on reenergization, suggesting a role for the MPT pore in loss of Ca^2+^‐homeostasis during reenergization (Rodrigo and Standen 2005a). Interestingly, cells that had hypercontracted but maintained Ca^2+^‐homeostasis also contracted in response to electrical stimulation, suggesting an intact cell membrane and E‐C coupling proteins.

Our study shows that rIPC was able to significantly delay the opening of MPT pore in response to oxidative stress of TMRE‐loaded myocytes to a similar level as conventional IPC, which suggests the central and common role of preventing MPT pore opening in protection by remote IPC and conventional IPC. A central signaling role for PKC*ε* in conventional IPC is well documented (Inagaki et al. 2006) and rIPC (Wolfrum et al. 2002) including an in vitro model of rIPC (Shimizu et al. 2009). We also show significant translocation of PKC*ε* in our model of rIPC myocytes comparable to the level in conventional‐IPC myocytes, and that inhibition of PKC*ε* blocks both cardioprotection and protection against opening of MPT pore in response to oxidative stress. Furthermore, this appears to involve NOS‐signaling downstream of PKC*ε*, as cardioprotection and the delay in MPT pore opening in rIPC‐myocytes is blocked by L‐NAME, whereas PKC*ε* translocation is still present (Figs. 7and 8). Prevention of MPT pore opening will prevent pore‐induced necrosis (Hausenloy et al. 2004; Halestrap 2006) and permit recovery and normal functioning of the mitochondria, and through the production of ATP assist in the maintenance of Ca^2+^‐homeostasis.

### Is conditioning of isolated myocytes with “conditioned perfusate” an appropriate model for remote preconditioning?

Przyklenk et al., first demonstrated that IPC applied to one coronary vascular bed conferred protection to the neighboring myocardium, leading to the suggestion that agents released from the ischemic myocardium were able to travel to and induce protection in naïve myocardium (Przyklenk et al. 1993). The presence of a humoral agent has since been implicated in rIPC involving ischemic myocardium (Dickson et al. 1999), mesenteric beds (Hajrasouliha et al. 2008), and skeletal muscle beds (Shimizu et al. 2009). Although recent studies have now shown the involvement of a neural component to rIPC, the presence of a humoral agent has since been implicated in rIPC involving mesenteric beds (Hajrasouliha et al. 2008) and skeletal muscle beds (Shimizu et al. 2009), and it is likely that rIPC involves both a neural and humoral component (Lim et al. 2010). In an extension of the initial study by Przyklenk et al., the group confirmed the presence of humoral agents release by ischemic myocardium, by demonstrating that the effluent from a preconditioned heart was able to confer protection to a naïve acceptor heart (Dickson et al. 1999). Our study adopts this latter model of collecting the effluent from IPC hearts, which is then applied naïve myocytes. The robustness of this observation is further highlighted by the ability of serum from rIPC humans to protect in vivo rabbit hearts and isolated myocytes against ischemia/reperfusion injury (Shimizu et al. 2009).

Evidence is growing to support the involvement of a neural pathway to rIPC (Lim et al. 2010; Merlocco et al. 2014) and a recent study combining pharmacological and gene knock‐down studies, suggest a crucial dependence of rIPC on vagal preganglionic neurones (Mastitskaya et al. 2012). The involvement of a neural pathway in itself does not rule out the generation of a blood borne agent, which likely involves neural signaling. Indeed, the generation of a humoral blood‐borne signal in response to rIPC, requires an intact femoral nerve (Steensrud et al. 2010) and a recent study showed that direct nerve stimulation results in cardioprotection through the release of humoral agents into the blood (Redington et al. 2012). Our study focuses on humoral agents released directly by the ischemic heart tissue, as any neural component is absent in the isolated Langendorff perfused heart. It is clear from our data that ischemia of the isolated rat heart results in the release of agent/s that are able to protect cardiomyocytes against reperfusion injury and this involves adenosine and opioid receptor activation.

### Study limitations

Our data show that conventional IPC results in inhibition of the NHE‐1 activity in the isolated myocytes, which contributes to the reduction in Ca^2+^‐loading during metabolic inhibition but that this increase is not a prerequisite of cardioprotection, which is dominated by an inhibition of MPT pore opening. However, it is well documented that an increase in [Ca^2+^]_i_ contributes to MPT pore formation during reperfusion (Javadov et al. 2003), and as IPC is known to exhibit a threshold in the induction of protection (Yellon and Downey 2003), it is likely that [Ca^2+^]_i_ could prove important when the preconditioning signals responsible for inhibiting MPT pore opening are reduced, that is, less cycles of IPC, diseased states such as diabetes (Balakumar and Sharma 2012).

Isolated myocytes offer a range of advantages over the intact heart in the drive to unravel the intricacies of the preconditioning pathway (Diaz and Wilson 2006). However, they differ significantly from myocytes in the myocardial wall, not only by the absence of surrounding endothelial and vascular smooth muscle cells, but also as they are not tethered mechanically to neighboring cells and are therefore less likely to undergo mechanical injury during a strong reperfusion‐induced hypercontracture driven by high [Ca^2+^]_i_ and ATP availability (Rodrigo and Standen 2005a; Garcia‐Dorado et al. 2012). So in an intact heart, hypercontracture, Ca^2+^‐loading and MPT pore opening are likely to have an integrated role in reperfusion injury and therefore the mode of protection from remote IPC in the intact heart through prevention of mechanical injury may indeed involve Ca^2+^‐regulation.

## Acknowledgments

Professor Nick Standen for his support, guidance, and inspiration over the years.

## Conflict of Interest

Sanglifehrin‐A was obtained from the Novartis Institutes for Biomedical Research, Basel, Switzerland.
